# Editorial: Management of neuroendocrine tumors of the head and neck

**DOI:** 10.3389/fendo.2025.1570791

**Published:** 2025-02-24

**Authors:** Anasuya Guha, Karel Pacak, Renato Mariani-Costantini, Jan Plzak, Ales Vicha

**Affiliations:** ^1^ Department of Otorhinolaryngology, Charles University, 3^rd^ Faculty of Medicine and University Hospital Kralovske Vinohrady, Prague, Czechia; ^2^ Section of Medical Neuroendocrinology, Eunice Kennedy Shriver National Institute of Child Health and Human Development, National Institutes of Health, Bethesda, MD, United States; ^3^ Center for Advanced Studies and Technology (CAST), Gabriele d’Annunzio University, Chieti, Italy; ^4^ Department of Otorhinolaryngology and Head and Neck Surgery, Charles University, 1^st^ Faculty of Medicine and University Hospital Motol, Prague, Czechia; ^5^ Department of Pediatric Hematology and Oncology, Charles University, 2^nd^ Faculty of Medicine and University Hospital Motol, Prague, Czechia

**Keywords:** neuroendocrine tumor, pituitary neuroendocrine tumor, head and neck neuroendocrine carcinoma, schwannoma of neck, head and neck paraganglioma (HNPGL), medullary thyroid cancer (MTC), genetic testing

Neuroendocrine tumors (NETs) of the head and neck are rare neoplasms with distinct clinical and pathological patterns; certain types also demonstrate hormonal activity or genetic predisposition. Neuroendocrine neoplasms account for <1% of head and neck malignancies and about 2% of neuroendocrine carcinomas (NECs). These are mainly categorized into two groups. Group I NETs are characterized by epithelial differentiation (1) and encompass a larger category of tumors, namely well-differentiated NECs (carcinoids), moderately differentiated NECs (atypical carcinoids) and poorly differentiated NECs (small and large cell carcinomas). In the head and neck, NECs mainly arise from the larynx, followed by the paranasal sinuses, salivary glands, and oropharyngeal mucosa. Rates of recurrence and metastasis as well as prognosis varies on the localization and grade of differentiation. Group II NETs are non-epithelial neurally-derived tumors (2); these include paragangliomas, schwannomas, and rarer tumors such as olfactory neuroblastomas, malignant peripheral nerve sheath tumors, and extra-osseous Ewing’s sarcomas.

Well differentiated NECs are usually located in the larynx and nearly 90% occur in the supraglottic region (2). Moderately and poorly differentiated small cell laryngeal tumors have higher rates of metastasis, local and distant respectively. Becht et al. reported a case of small cell carcinoma in the subglottic region with cervical lymph node metastasis. Radical sequential chemoradiotherapy was administered. The patient presented a year later with metastasis in multiple sites. Palliative therapy had little effect. Although, first line chemotherapy using Cisplatin and Etoposide showed good response, nonetheless, it was evident that refractory disease presents a considerable challenge. The authors recommended more precise and personalized diagnosis along with the development of novel therapeutic approaches.

Pituitary NETs (PitNETs), previously known as pituitary adenomas (1) consist of functional (hormone secreting) and nonfunctional tumors (3). Patients may be asymptomatic, otherwise exhibit clinical syndrome due to hormone hypersecretion or compression of adjacent structures. In a study of patients with pitNETs, Hu et al. found both reduced and elevated IGF-1 levels were strongly associated with an increased risk of nonalcoholic fatty liver disease (NAFLD) compared with those with normal IGF-1 level. This increases the risk of liver hypertrophy impairing glucose and lipid metabolism, as well as associated comorbidities and mortality. The article emphasizes the importance of maintaining IGF-1 in an optimal range to prevent NAFLD in PitNET patients. Interestingly, Zhu et al. demonstrated a rare variant of an ectopic thyroid-stimulating hormone (TSH) tumor located in the nasopharynx. Symptoms of thyrotoxicosis and elevated levels of TSH were noted. A 68Ga-DOTATATE PET/CT scan showed increased DOTATATE uptake, and an octreotide suppression test showed reduction in TSH levels. Tumor histochemistry showed positive markers for an ectopic pituitary NET. Therefore, diagnosis of an ectopic pituitary tumor should be considered in cases with hyperthyroidism and raised TSH levels. Furthermore, Lamas et al. studied the efficacy and safety of Temozolomide (TMZ) in the treatment of aggressive PitNETs, which are uncommon variants (3). TMZ is currently recommended as first-line therapy by the current guidelines of the European Society of Endocrinology for such tumors after failed standard therapies (4). Despite the subsequent risk of tumor progression (36.4%), TMZ proved an effective medical treatment, with up to 81.5% tumor shrinkage or stabilization. The study concluded that overall progression free survival was higher in patients treated with concomitant radiotherapy, although more definitive treatment protocols are needed.

Medullary thyroid carcinoma (MTC) originates from parafollicular C cells, that characteristically secrete Calcitonin. In majority of cases, MTCs occur sporadically, whilst 20% are inherited as part of the multiple endocrine neoplasm spectrum (5). Chakraborty et al. showed a relationship between RET mutation positive MTCs and female preponderance, not reported previously. According to the authors, Calcitonin level evaluation allows preoperative diagnosis of sporadic MTCs in cases of nodular thyroid disease. In this context, it should be considered that incidence of neck node metastasis is high in both MTCs and atypical moderately differentiated NECs of the larynx. These also share histological similarities and both may produce calcitonin.

Schwannomas are benign neoplasms originating from Schwann cells of the neural sheaths of motor and sensitive peripheral nerves, with 25-45% located in the extracranial head and neck region (6). Uptake of ^99m^Tc-sestamibi in the localization of acoustic neuromas is known, although not been used to locate schwannomas of the neck. Fiore et al. described an incidental radiological finding of a neck mass. SPECT/CT with ^99m^Tc-sestamibi avidity initially raised suspicion of a nonfunctional parathyroid mass or hyperplastic thyroid nodule. Diagnosis of a schwannoma was made on core needle biopsy. Suspected origin was the left recurrent laryngeal nerve, however intraoperative and histopathological findings confirmed an esophageal submucosal schwannoma, a rare occurrence. This finding suggests that schwannomas need to be considered in the differential diagnosis of cervical masses with focal 99mTc-sestamibi uptake.

Head and neck paragangliomas (HNPGLs) are classified as mostly benign vascular tumors originating from paraganglial cell clusters (1, 7). These vary in anatomical locations. The disease can occur in a sporadic or hereditary form. Amongst all the genes, the Succinate Dehydrogenase subunit D (*SDHD)* gene, has been proven to have the highest affinity for HNPGLS (7). The *SDHD* gene mutation is also found in the absence of positive family history, thus giving rise to occult familial cases. Guha et al. found that, in contrast to previously published large-scale studies, only one-third of Czech patients with known family history showed germline pathogenic *SDHD* mutation; nearly 78% of these patients could be described as occult familial cases. Furthermore, *SDHD* mutation was found in only 12% of the Czech patients. A common *SDHD* mutation variant was shared amongst unrelated patients; nonetheless no founder-effect was established. The authors reiterated the importance of genetic testing in the management of HNPGL patients.

The Research Topic portrays an array of differential diagnosis, diagnostic techniques and therapeutic options. It is evident that NET patients require multidisciplinary management with a personalized approach. The lack of evidence in the literature primarily stems from the rarity of the disease. Other major issues include the cost-effectiveness and availability of diagnostic tools, as well as lack of clinical expertise, therapeutic measures and clinical trials in medical facilities. Therefore, even with limited number of cases, certain steps such as maintaining a central patient registry, sharing clinical experiences and consulting with experts, promoting further research, additionally expanding global collaborations thus creating international consensus could moderately ease the challenges faced in the management of such patients.

## References

[1] Mete O Wenig BM. Update from the 5th edition of the world health organization classification of head and neck tumors: overview of the 2022 WHO classification of head and neck neuroendocrine neoplasms. Head Neck Pathol. (2022) 16:123–42. doi: 10.1007/s12105-022-01435-8 PMC9018952 35312985

[2] Subedi N Prestwich R Chowdhury F Patel C Scarsbrook A. Neuroendocrine tumours of the head and neck: anatomical, functional and molecular imaging and contemporary management. Cancer Imaging. (2013) 13:407–22. doi: 10.1102/1470-7330.2013.0034 PMC3830426 24240099

[3] Trouillas J Jaffrain-Rea M-L Vasiljevic A Raverot G Roncaroli F Villa C. How to classify pituitary neuroendocrine tumors (PitNET)s in 2020. Cancers (Basel). (2020) 12:514. doi: 10.3390/cancers12020514 32098443 PMC7072139

[4] Raverot G Burman P McCormack A Heaney A Petersenn S Popovic V. European Society of Endocrinology Clinical Practice Guidelines for the management of aggressive pituitary tumours and carcinomas. Eur J Endocrinol. (2018) 178:G1–24. doi: 10.1530/EJE-17-0796 29046323

[5] Rendl G Manzl M Hitzl W Sungler P Pirich C. Long-term prognosis of medullary thyroid carcinoma. Clin Endocrinol (Oxf). (2008) 69:497–505. doi: 10.1111/j.1365-2265.2008.03229.x 18331612

[6] Shrikrishna BH Jyothi AC Kulkarni NH Mazhar M. Extracranial head and neck schwannomas: our experience. Indian J Otolaryngol Head Neck Surger. (2016) 68:241–7. doi: 10.1007/s12070-015-0925-5 PMC4899365 27340644

[7] Guha A Musil Z Vicha A Zelinka T Pacak K Astl J. A systematic review on the genetic analysis of paragangliomas: Primarily focused on head and neck paragangliomas. Neoplasma. (2019) 66:671–80. doi: 10.4149/neo_2018_181208N933 PMC6826254 31307198

